# Purification and biofabrication of 5-aminolevulinic acid for photodynamic therapy against pathogens and cancer cells

**DOI:** 10.1186/s40643-022-00557-9

**Published:** 2022-06-16

**Authors:** Yen-Ju Lee, Ying-Chen Yi, Yu-Chieh Lin, Chao-Chung Chen, Jia-Horung Hung, Jia-Yi Lin, I-Son Ng

**Affiliations:** 1 Department of Chemical Engineering, National Cheng Kung University, Tainan, Taiwan; 2 Institute of Clinical Medicine, College of Medicine, National Cheng Kung University, Tainan, Taiwan; 3 Department of Ophthalmology, National Cheng Kung University Hospital, College of Medicine, National Cheng Kung University, Tainan, Taiwan

**Keywords:** 5-Aminolevulinic acid, Ion exchange chromatography, Photodynamic therapy, Antimicrobial, Cancer cell, Microalgae

## Abstract

5-Aminolevulinic acid (5-ALA) is a non-proteinogenic amino acid which has involved in heme metabolism of organisms, and has been widely applied in agriculture, and medical fields nowadays. 5-ALA is used in the elimination of pathogens or cancer cells by photodynamic therapy (PDT) owing to the photosensitizer reaction which releases the reactive oxygen species (ROS). Currently, biofabrication of 5-ALA is regarded as the most efficient and eco-friendly approach, but the complicated ingredient of medium causes the nuisance process of purification, resulting in low recovery and high producing cost. In this study, hydrogen chloride, sodium acetate, and ammonia were examined to maximize the recovery of 5-ALA from ion-exchange chromatography (IEC), thus a 92% recovery in 1 M ammonia at pH 9.5 was obtained. Afterward, the activated carbon was used for decolorization to further remove the pigments from the eluent. Four organic solvents, i.e., diethyl ether, methanol, ethanol, and acetone were compared to extract and form 5-ALA precipitation. The purified 5-ALA was verified to eliminate 74% of A549 human lung cancer and 83% of A375 melanoma skin cancer cell. Moreover, *Proteus hauseri*, *Aeromonas hydrophila*, *Bacillus cereus*, and *Staphylococcus aureus* were killed via anti-microbial PDT with 1% 5-ALA and reached 100% killing rate at optimal condition. With the addition of 0.05% 5-ALA during the culture, the growth of microalgae *Chlorella sorokiniana* was improved to against a common aquatic pathogen, *A. hydrophila*. The broad application of 5-ALA was demonstrated in this study for the first time.

**Graphical Abstract:**

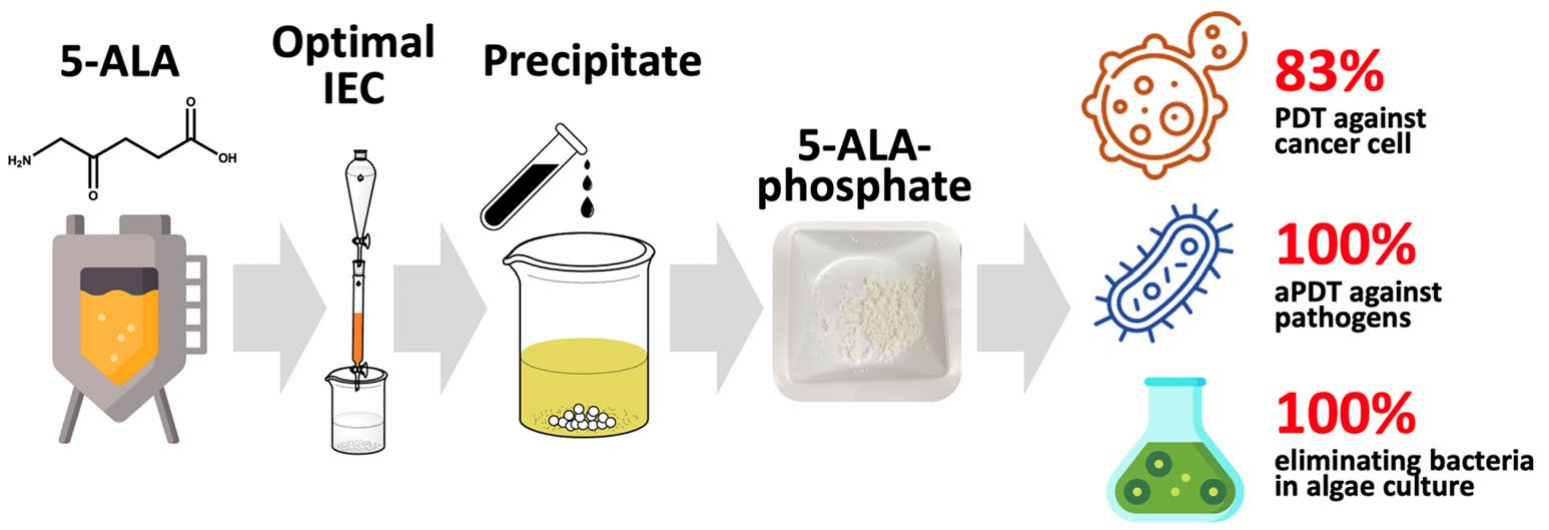

## Introduction

5-Aminolevulinic acid (5-ALA), an endogenous, non-proteinogenic amino acid, is a precursor in the biosynthesis of all porphyrins and tetrapyrrole compounds (Yi et al. 2021a). In recent decades, 5-ALA with different purity has wide applications in agricultural (Hotta et al. 1997) and the medical field (Inoue 2017; Juzeniene et al. 2002). 5-ALA can be used as a growth promoter or insecticide depending on its concentration. A low concentration of 5-ALA stimulates cell metabolism and assists the growth of plants and crops (Sasaki et al. 2002). On the contrary, harmful insects can be eliminated with a higher amount of 5-ALA on plants when the insects consume the 5-ALA. The pure 5-ALA has been used as a precursor of photosensitizer in photodynamic therapy (PDT) to diagnose tumor cells, treat cancer cells and cure skin diseases (Yi et al. 2021a). In this therapy, a photosensitizer protoporphyrin IX (PPIX) was produced and showed fluorescence when a high concentration of 5-ALA accumulated in the cell. PPIX would cause reactive oxygen species (ROS) under appropriate wavelength and lead to cell damage by generating singlet oxygen. 5-ALA-induced photodynamic therapy and diagnosis had been reported since 1990 (Kennedy et al. 1990) and were approved by FDA in 2017. Moreover, 5-ALA can be applied in anti-microbial PDT (aPDT) to attain non-invasive and non-toxic treatment for various wound infections.

The antibacterial photodynamic therapy (aPDT) has been recently demonstrated as one of the most outstanding approaches to treating multi-drug resistant (MDR) pathogens, containing three important elements: photosensitizer (PS), illuminating light with fitting wavelength, molecular oxygen. As 5-ALA is converted into PPIX, a strong PS, the accumulated PPIX in the cell will cause apoptosis by producing ROS under the illumination by red light (Yi et al. 2021b). Since both 5-ALA and PPIX are natural metabolites in organisms, 5-ALA-aPDT is an effective and non-toxic treatment to all kinds of pathogens and MDR. Thus, 5-ALA is a valuable and potential chemical compound in this era.

5-ALA is produced from chemical synthesis or biofabrication. However, the chemical synthesis of 5-ALA is in low yield and high cost. Moreover, toxic compounds will be released during the production of 5-ALA via chemical process (Matsumura et al. 1994; Miyachi et al. 1998). In contrast, a high-yield 5-ALA can be achieved via biosynthesis in an eco-friendly mode, making biotransformation of 5-ALA a fascinating approach. However, the saccharides, protein, amino acids, organic acids, and metal ions retained in the 5-ALA fermentation reduce the efficiency of 5-ALA purification and decrease the antibacterial activity (Okada et al. 2016). Therefore, purification of 5-ALA from the broth is critical and necessary. Generally, the 5-ALA in microbial broth has been purified by using ion-exchange chromatography (IEC) from other compounds and contaminants (Venosa et al. 2004; Din et al. 2021). The optimal condition for desorption of different compounds on the resin in IEC may differ from the distinct properties. Moreover, the ingredients in the culture medium and the additional substrates for 5-ALA production make the separation of 5-ALA in IEC difficult. Therefore, the pH value, ion strength, and the isoelectric point of the compound should be optimized for a high desorption rate in IEC.

In this study, 5-ALA was purified by using IEC from fermentation broth. To maximize the recovery of 5-ALA, the eluent buffer, ion concentration and pH were optimized through desorption process from chromatography. After preliminary purification by IEC, 5-ALA solution was adjusted by phosphoric acid to pH 3 and stirred with activatied carbon to remove the colored molecules. The rotary evaporator was employed to obtain a higher concentration of 5-ALA before precipitation. Finally, 5-ALA was precipitated with diethyl ether, methanol, ethanol, and acetone. The purified 5-ALA was applied to against 2 different tumor cells and 4 pathogens by PDT, and eliminated *Aeromonas hydrophila* (*A. hydrophila*) in algal culture to examine the antimicrobial ability of 5-ALA.

## Materials and methods

### Chemicals

Amberlite^®^IR120 strong acid cation exchange resin, 5-ALA and acetylacetone were purchased from the Sigma-Aldrich, USA. Hydrochloride acid was purchased from Fluka, Switzerland. Ammonium hydroxide was purchased from Thermo-Fisher, USA. Activated carbon was ordered from Alfa Aesar, USA. Phosphoric acid was purchased from Merck, USA. Acetone, diethyl ether, ethanol, and methanol were purchased from ECHO, Taiwan. 4-Dimethylaminobenaldehyde (DMAB) was ordered from ACROS Organics™. Perchloric acid and sodium acetate were purchased from SHOWA, Japan.

### Culture condition of 5-ALA

Biofabrication of 5-ALA was carried out by culturing strain RcI from a previous publication (Yu et al. 2022). RcI was precultured in Luria–Bertani (LB) medium at 37 °C, 200 rpm for 16 h. The preculture cell was inoculated with 2% (*v/v*) into 300 mL MM9 medium containing (NH_4_)_2_SO_4_ (16 g/L), Na_2_HPO_4_•12H_2_O (16 g/L), KH_2_PO_4_ (3 g/L), yeast extract (2 g/L), MgSO_4_•7H_2_O (0.5 g/L), MnSO_4_•7H_2_O (0.01 g/L), glucose (20 g/L), and glycerol (10 g/L) in a 1-L bioreactor at 37 °C, 300 rpm with 1 *vvm* aeration. The final concentration of 0.1 mM IPTG, 0.4 mM ferric citrate, 4 g/L glycine, 1 g/L succinate and 30 μM PLP were added in cultivation when OD_600_ reached 0.6–0.8 and shifted the culture to 30 °C and 500 rpm until 24 h. Substrates including 3 g/L glycine, 1.5 g/L glucose, and 2 g/L succinate were fed at 12 h. The cell concentration was measured by a spectrometer (SpectraMax 340, Molecular Devices, USA) with an optical density at 600 nm (OD_600_).

### Ehrlich assay for quantification of 5-ALA

A 200 μL 5-ALA sample was mixed with 200 μL sodium acetate (pH 4.6) and 40 μL acetylacetone, then the mixture was heated at 100 °C for 10 min to accelerate the reaction (Yu et al. 2022). After cooling to room temperature, the mixture was mixed and reacted with the same volume of Ehrlich’s reagent for 10 min in dark. Finally, the solution was analyzed by optical density at wavelength 553 nm by using a spectrophotometer.

### Purification of 5-ALA using chromatography

The strong acid cation exchange resin (Amberlite^®^ IR120) was packed in a column (7.07 cm^2^, 20 cm height). First, the resin was immersed in 50 mL of 1.5 M HCl for 1.5 h, followed by 50 mL of 1.5 M NaOH. A 50 mL of 1.5 M HCl was passed through the column to prepare an H^+^-form condition. The resin was washed by ddH_2_O once between each step. The culture broth of 5-ALA was adjusted to pH 4.2–4.8 with acetate acid before adsorption. A 600 mL broth was applied to the column and then 100 mL ddH_2_O passed through to wash out the residual medium. HCl, sodium acetate buffer (SAB) and ammonia were applied in this study to examine the efficiency of 5-ALA desorption with different concentrations and different pH. Finally, 85% phosphate acid was added into the desorbed 5-ALA solution and adjust the pH to 3.0.

### Crystallization of 5-ALA

To remove the impurities in the broth, different amount of activated carbon was added into the solution and stirred at 500 rpm for 30 min for decolorization. The solution was then concentrated in a rotary evaporator to obtain a higher concentration of 5-ALA (i.e., 250 to 500 g/L), which was dripped into the different organic solvents including diethyl ether, methanol, ethanol, or acetone (Tachiya 2016). Finally, the precipitate was dried in the vacuum dryer (EYELA, Japan).

### HPLC analysis

The high-performance liquid chromatography (HPLC, Hitachi, Japan) was employed to analyze the purity of 5-ALA precipitate. Derivatization of samples were performed by the reaction consisting of 680 μL of 0.05 M borate buffer (pH 9), 480 μL of 100% methanol, 12 μL sample and 30 μL of 200 mM diethyl ethoxymethylenemalonate (DEEMM). The samples were heated at 70 °C for 2 h to complete the degradation of excess DEEMM and derivatization. Afterward, the samples were placed into HPLC with a quaternary pump, an inline degasser, an autosampler, and a column thermostat. Chromatographic separation was carried out by reverse-phase chromatography on a C18 column (YMC-C18 column, 4.6 × 250 mm, 5 μm particle size), maintained at 35 °C. Mobile phase A was composed of 100% acetonitrile, and B was made up of 25 mM aqueous sodium acetate buffer (pH 4.8). The flow rate of 0.8 ml/min was used, with the following gradient program: 0–2 min, 20–25% A; 2–32 min, 25–60% A; 32–40 min, 60–20% A. Detection was carried out at 284 nm (Xue et al. 2020).

### Cancer cell culture and photodynamic therapy

Human lung adenocarcinoma cells (A549 cells) and melanoma skin cancer cells (A375 cells) were purchased from Bioresource Collection and Research Center (BCRC, Taiwan), and cultured in Dulbecco’s modified Eagle’s medium (DMEM, Gibco, Grand Island, NY, USA) supplemented with 10% (*v/v*) fetal bovine serum (FBS, Invitrogen, Carlsbad, CA, USA). All cells were incubated in 10-cm tissue culture dishes at 37 °C and 5% (*v/v*) CO_2_. The cancer cells were seeded in 96-well plates using a fresh DMEM culture medium, then incubated under 37 °C and 5% (*v/v*) CO_2_ for 24 h before being treated by 5-ALA-PDT. The cells were incubated for 3 h with different concentrations of 5-ALA (0, 5, 10 g/L). Thereafter, the cells were exposed to the red light source at 635 nm with power density 100 J/cm^2^ for 15 min (HUA YANG Precision Machinery Co., Taiwan). After PDT treatment, the cells were incubated at 37 °C and 5% (*v/v*) CO_2_ for different time (0, 2, 4, 12 h) to make reactive oxygen species attack cells. Finally, the CCK-8 assay was applied to identify the viability of the cells. Before performing the cell counting kit-8 (CCK-8) assay, the culture medium consisting of 5-ALA was removed due to the background value. 10 µL of the CCK-8 reagent (MedChemExpress Ltd.) and 100 µL of the DMEM were added to each well, incubated the cells were at 37 °C and 5% (*v/v*) CO_2_ for 1 h, and optical density at 450 nm was measured using a spectrophotometer. Statistical analysis was performed using GraphPad Prism software version 8.0 (GraphPad Prism software, San Diego, USA). Differences in cell viability among the groups were analyzed using a t-test, and the values of *p* < 0.05 were statistically significant.

### Antibacterial photodynamic therapy (aPDT) against pathogens

The elimination of *P. hauseri* by aPDT was carried out with minor modifications from a previous study (Yi et al. 2021b). The pathogen was incubated in a 10 mL LB medium at 37 °C and 175 rpm for 16 h. The concentration of *P. hauseri* was adjusted to OD_600_ at 0.2 (approximately 10^9^ cells/mL) and injected 180 µL into 96-well plates. An appropriate amount of 5-ALA solution (i.e., 0.25%, 0.5% and 1%) was added to the cell sequentially. The plates were wrapped with aluminum foil to avoid the light and incubated at 37 °C for 3 h to metabolize 5-ALA to PPIX. Subsequently, it was illuminated with or without LED red light at a wavelength of 635 nm for 30 min, corresponding to power density of 100 J/cm^2^ for aPDT (HUA YANG Precision Machinery Co., Taiwan). Finally, to calculate the colony-forming unit (CFU), a 20-µL aliquot with a dilution rate of 10^–1^ to 10^–5^ was spread onto an agar plate and incubated at 37 °C for 16 h. The *A. hydrophila, Bacillus cereus* (*B. cereus*)*, Staphylococcus aureus* (*S. aureus*) were tested by following the procedure as aforementioned. Each experiment was carried out in triplicate.

### aPDT in the algal culture

*Chlorella sorokiniana* (Cs) was precultured in TAP medium for 2 days to reach OD_680_ at 0.8 (Lin et al. 2021). *A. hydrophila* was precultured in LB medium at 37 °C, 200 rpm for 16 h. The mixture of 5-ALA, *A. hydrophila* and Cs were prepared in a 10 mL TAP medium and cultured under white light (100 µmol/m^2^/s) with shaking at 150 rpm. The final_600_ concentration was adjusted to OD_680_=0.08 for Cs, and 0.2, 0.3, or 0.4 of OD_600_ for *A. hydrophila,* while the 5-ALA concentrations were used by 0.05%, 0.1% and 0.2%, respectively. Afterward, a 20-µL aliquot was dropped on the TAP plate for a 3-day cultivation to observe the growth of Cs and *A. hydrophila*.

## Result and discussion

### Optimization of chromatography for 5-ALA

**Fig. 1:**
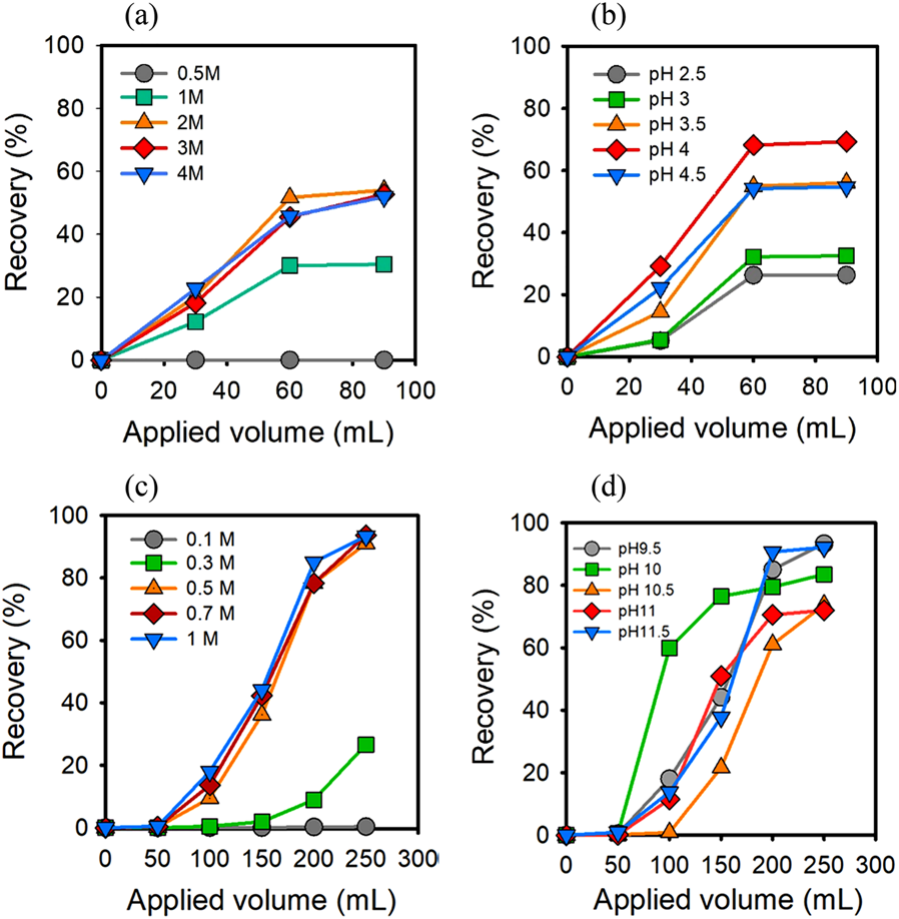
Effect of sodium acetate (**a**, **b**) and ammonia (**c**, **d**) for elution of 5-ALA from IEC. Recovery by using (**a)** different concentration of sodium acetate at pH 3.5, and (**b)** 2 M CH_3_COONa with different pHs. Recovery by using (**c)** different concentration of ammonia at pH 11 and (**d)** 1 M NH_4_OH with different pHs

To maximize the recovery of 5-ALA from the desorption step, the desorption efficiency of HCl, sodium acetate buffer (SAB) and ammonia were compared. As shown in Table 1, the lowest recovery of 12.1% was acquired by 3 M HCl while the 92% recovery was achieved by 1 M ammonia at pH 11.5 among all conditions. Since 5-ALA was adsorbed on the strong acid cation resins, the alkaline eluents could reduce the affinity of substrate and exchange 5-ALA from the resin (Moreira and Gando-Ferreira 2012). Afterwards, the optimal recovery of SAB and ammonia in different pH and concentrations were examined and shown in Fig. 1. The high concentration of both SAB and ammonia reached high recovery, which may result from the intensive molecular collision of cation and the adsorption site. However, the recovery remained 60% with 2 M to 4 M SAB (Fig. 1a), while 85 ± 5% recovery was accomplished by 0.5 M to 1 M ammonia (Fig. 1c). Patrickios and his colleagues reported that the chemicals are electrically neutral and the affinity between resins and molecular are lost at isoelectric point, enabling the acquisition of compounds from IEC (Patrickios and Yamasaki 1995). 5-ALA was more stable in an acidic surrounding (pH 2—4) than that in the alkaline condition (Bunke et al. 2000), but the alkaline eluent was more favorable for its purification from IEC due to the isoelectric point at pH 5.69. When ammonia was applied, 5-ALA possessed negative charges and left the resin at pH higher than 5.69 in this study. Therefore, 92% of 5-ALA recovery was carried out by 1 M ammonia at pH 9.5 (Fig. 1c, d), which was 1.3-folds higher than using 1 M sodium acetate buffer at pH 4.67 and followed by pH 3.8 condition in the previous report (Tripetch et al. 2013).

**Table 1:** The absorption, desorption, and recovery of 5-ALA from cation ion-exchange chromatography using different eluents

Eluents	Conc. (M)	pH	Applied volume (mL)	Adsorbed^a^ 5-ALA (g)	Desorbed 5-ALA (g)	Recovery (%)
HCl	1.5	ND	110	1.24	0.101	8.1
HCl	3.0	ND	110	1.24	0.149	12.0
CH_3_COONa	0.74	3.1	110	1.24	0.78	62.9
CH_3_COONa	0.74	3.1	250	1.24	0.79	63.7
NH_4_OH	1.0	11.5	250	1.25	1.15	92.0

^a^All the chromatography is injected by 300 mL 5-ALA at 4.16 g/L for each batch. ND means not determined

### Decolorization, crystallization and purity of 5-ALA

**Fig. 2:**
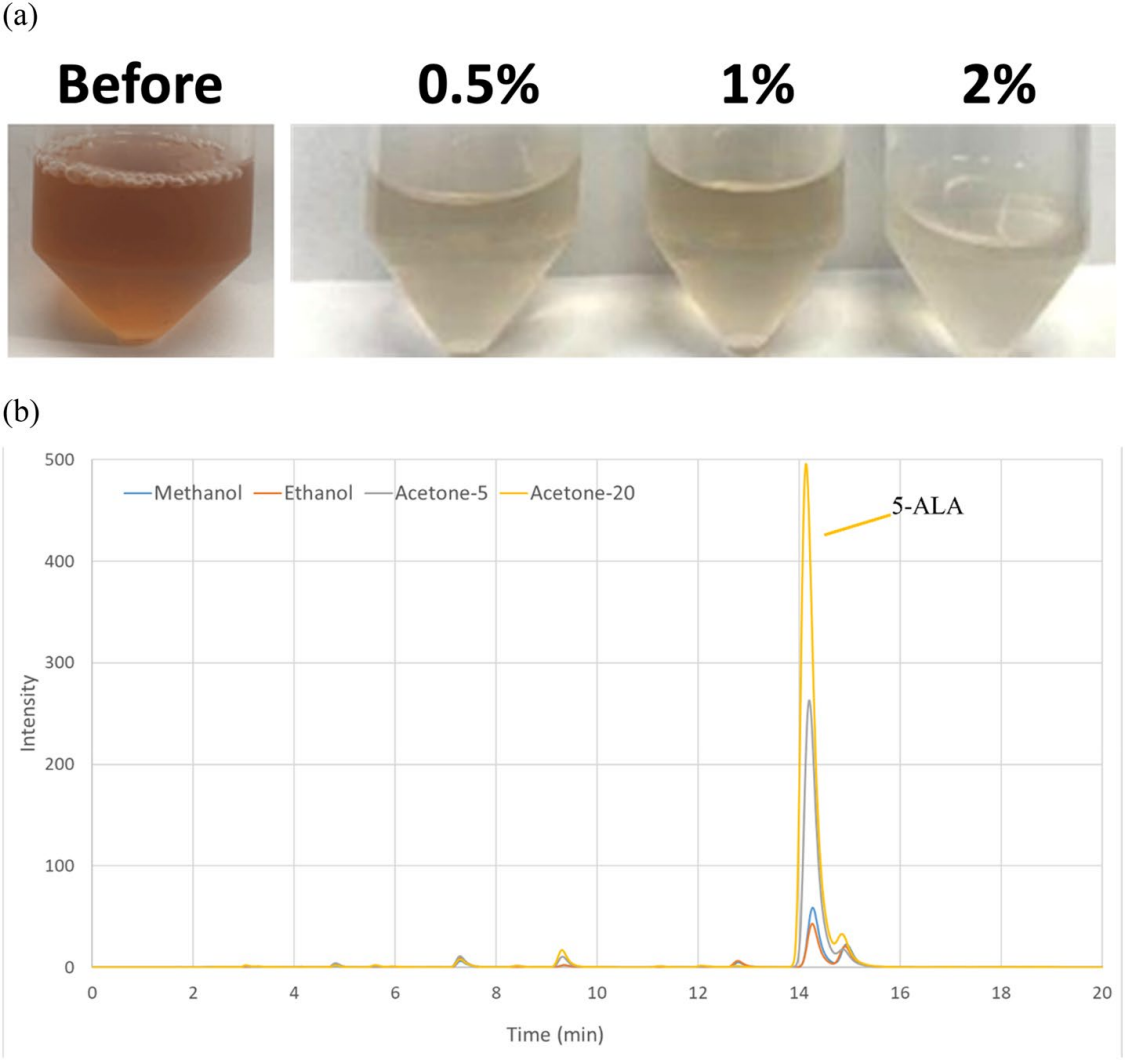
**(a)** Decolorization of 5-ALA after IEC using 0.5%, 1% and 2% activated carbon. (**b)** The HPLC result for 5-ALA precipitation from different organic solvents including methanol (10:1), ethanol (10:1), acetone (5:1) and acetone (20:1). The volume ratio of solvent to 5-ALA (*v/v*) is shown in the parentheses

As the pigments in the fermentation broth would decrease the purity of 5-ALA during the process, a different amount of activated carbon was employed for decolorization. The solution after decolorization with 0.5%, 1% and 2% activated carbon was clear as shown in Fig. 2a. Subsequently, the solution was adjusted to pH 3 with 85% phosphoric acid for 5-ALA stability. To obtain a high concentration of 5-ALA up to 500 g/L before crystallization, the solution after decolorization was concentrated by a rotary evaporator at 65 °C. Afterward, the poor solvents: diethyl ether, methanol, ethanol, and acetone, were used in the crystal method and dehydrated the 5-ALA solution. The result indicated that the ketones and alcohols were better solvents for dehydration of 5-ALA (Table 2). The most precipitates were obtained from acetone with a volume ratio of 10:1, reaching 50% purity of 5-ALA. However, there was only oily liquid occurred in the solution and no precipitates from diethyl ether. As shown in HPLC analysis (Fig. 2b), acetone was the best solvent for 5-ALA precipitation when a higher concentration of purified 5-ALA was obtained at the volume ratio of 20:1 (acetone/5-ALA).

**Table 2:** The purity of 5-ALA from different solvents extraction and precipitation

Organic solvents	Solvent volume (mL)	5-ALA volume (mL)	Crude product (g)	5-ALA content (g)	Purity (%)
Diethyl ether	20	2	0.001	ND	ND
Methanol	20	2	0.12	0.005	4.17
Ethanol	20	2	0.30	0.007	2.33
Acetone	10	2	0.36	0.13	36.1
Acetone	20	2	0.50	0.25	50.0
Acetone	40	2	0.45	0.21	46.7

^a^5-ALA is used at concentration of 500 g/L. ND means not determined

### 5-ALA-PDT treatment on A549 and A375 cancer cells

**Fig. 3:**
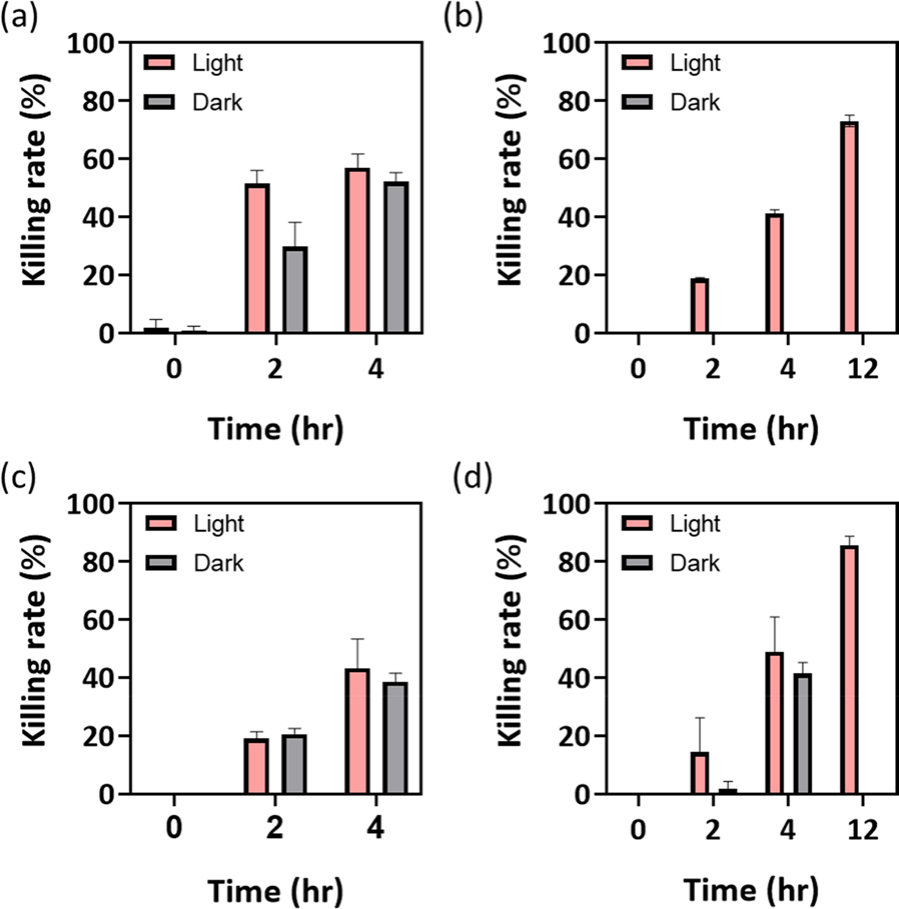
Cell viability with purified 5-ALA treatment among different incubated time. Killing rate of (**a)** 0.5% and (**b)** 1% 5-ALA treatment to A549 human lung cancer cells. Killing rate of (**c)** 0.5% and (**d)** 1% 5-ALA treatment to A375 melanoma skin cancer cells

In this section, we focused on the effectiveness of 5-ALA-PDT for cancer cells, A549 and A375 cells. When treated with 5-ALA-PDT, the viability of the cells showed differences between the cells incubated with increasing concentrations of 5-ALA from 0.5 to 1 g/L, and the control group (*p* < 0.0001). As the incubation time increased, the inhibition of both cancer cells also increased, and the enhancement in killing rate with time was more significant when 1% 5-ALA was used and shown in Fig. 3 (*p* = 0.0053 for A549 cells and *p* < 0.001 for A375 cells). Cell viability was significantly lower in the treatment groups than in the control groups under 1% 5-ALA treatment. As a result, 56% and 43% killing rates for A549 cells and A375 cells were obtained by 5-ALA-PDT treatment with 0.5% purified 5-ALA under 4 h incubation. After increasing to 1% 5-ALA, the cell killing rate reached 74% (*p* = 0.0003) and 83% (*p* = 0.0002) under 12 h incubation for A549 cells and A375 cells, respectively (Fig. 3b, d). Previous studies have suggested the potential of 5-ALA-PDT in treating cancer cells and the inhibition of cell survival activity significantly depend on both dose and time (Cai et al. 2018; Teijo et al. 2016; Wachowska et al. 2011). The results verified the cell necrosis of A549 and A375 cells when 5-ALA was administered externally under light exposure or dark.

### Antibacterial activity against pathogens using aPDT

Wound infection is a perilous defect of skin or soft tissue caused by pathogenic organisms which triggers the immune response in human bodies. Depending on the etiology and severity of the microbial invasion, the infections range from minor superficial to fatal symptoms (Simões et al. 2018). Traditionally, antibiotics such as beta-lactams, glycopeptides, quinolones, sulphonamides and tetracyclines were developed for medical applications to eliminate the pathogens for wound recovery. Among all the antibiotics, penicillin has been the most prevalent against various pathogens in wound infection (Shukla et al. 2020). Penicillin was a revolutionary discovery to fight against bacterial infections in the first half of the twentieth century, and the antibiotics developed from penicillin reduced the mortality caused by infectious diseases effectively (Sarmah et al. 2018). However, the misuse and abuse had a serious consequence: the generation of multi-drug resistant (MDR). According to WHO’s estimations, approximately 700,000 deaths are caused by MDR infections every year (Klausen et al. 2020). Owing to the problems above, the aPDT is known as a new therapeutic assay.

Four pathogens, *P. hauseri*, *A. hydrophila*, *B. cereus*, and *S. aureus*, were selected for 5-ALA-aPDT in this study. *Proteus* species are widespread in the environment which causes the urinary tract infections (UTIs) in human by spreading from the rectum to the periurethral and bladder (Armbruster and Mobley 2012). *A. hydrophila* is one of the most common bacteria isolated in freshwater, seawater, and sewage environments. In addition, *A. hydrophila* is the cause of zoonotic diseases such as gastrointestinal disease, sepsis, and aquatic wound infection (Kussovski et al. 2009). *B. cereus* is associated with foodborne illness and food spoilage, provoking vomiting, and diarrhea to humans (do Prado-Silva et al. 2021). *S. aureus* is the most epidemic pathogen, which is often found in human skin, mucous membranes, and purulent wounds, causing vomiting, abdominal pain, diarrhea, and fever (Pérez-Laguna et al. 2018).

**Fig.4:**
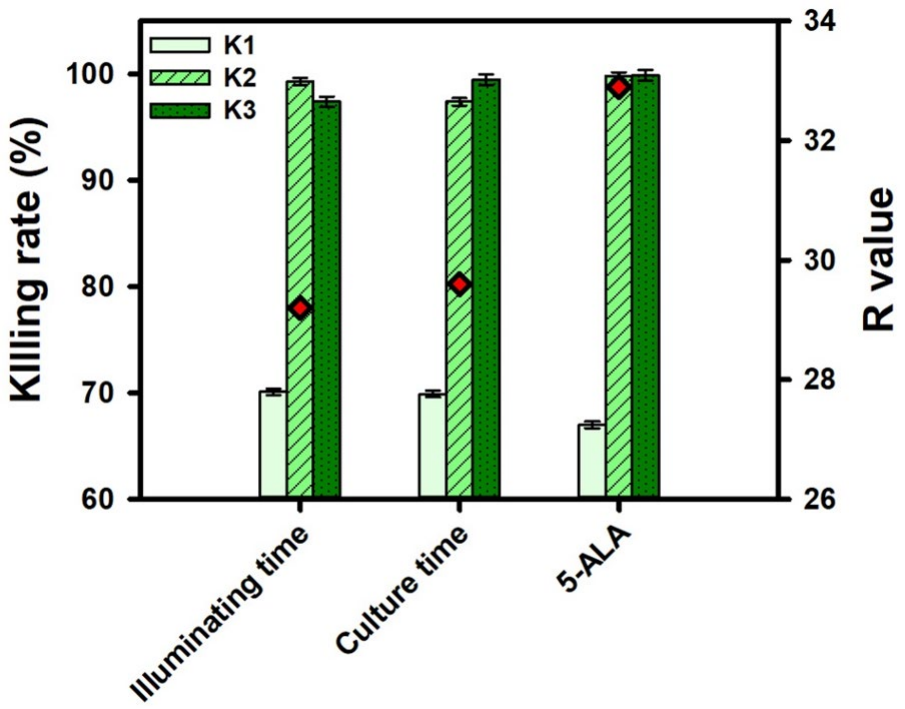
Taguchi L9 experimental results for 5-ALA aPDT against the pathogen of *Proteus hauseri.* K1, K2 and K3 mean the average of killing rate for 3 levels of each parameter. The three levels of each factor: illuminating time (15, 30, 45 min); culture time (2, 3, 4 h); 5-ALA concentration (1%, 2%, 3%). R is the divergence which is indicated by red dot

To optimize the condition of 5-ALA-aPDT, the Taguchi L9 method was designed with 3 factors: illuminating time, culture time and 5-ALA concentration. The experiments were carried out on *P. hauseri* and the efficiency of each group was shown in Table 3. The K values indicated that the optimal illuminating time was 30 min (Fig. 4). Since  5-ALA would be degraded under strong light exposure, resulting in low killing rate. On the other hand, the killing rate showed a positive correlation with culture time and 5-ALA concentration. From the R-value, the concentration of 5-ALA was the dominant factor in 5-ALA-aPDT treatment, while the illuminating time and culture time had similar influences on the result. Therefore, the optimal condition for the elimination of *P. hauseri* by 5-ALA-aPDT was acquired by 3% of 5-ALA with 30-min illumination and 4-h cultivation. Although it showed better efficiency of aPDT with higher concentration of 5-ALA (i.e., 2 to 3%) from the Taguchi’s L9 experiment, using 1% of 5-ALA still eliminated *P. hauseri* effectively. Taking the cost effect on the process, using 1% of 5-ALA was economically efficient to further optimize antibacterial activity.

**Table 3:** Taguchi L9 experiment design for optimization of 5-ALA aPDT against the pathogen of *Proteus hauseri*

No.	Illumination time (min)	Culture time (h)	5-ALA conc. (%)	Killing rate (%)
1	15	2	1	10.3
2	15	3	2	100
3	15	4	3	100
4	30	2	2	99.5
5	30	3	3	99.9
6	30	4	1	98.4
7	45	2	3	99.8
8	45	3	1	92.3
9	45	4	2	100

**Fig. 5:**
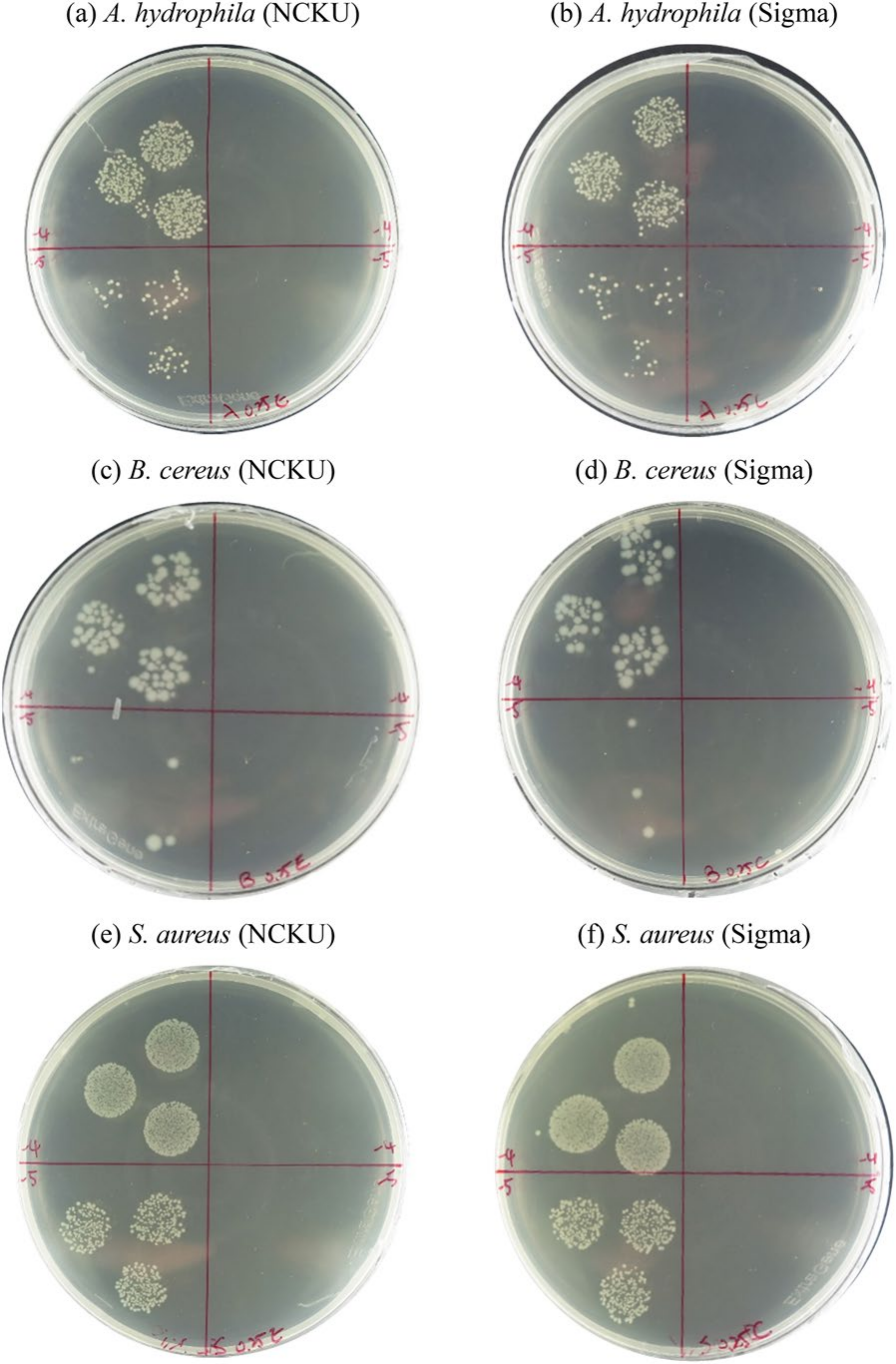
The plate assay of aPDT using 5-ALA from (**a**, **c**, **e**) purified (NCKU) and (**b**, **d**, **f**) commercial (Sigma A3785) against pathogen *Aeromonas hydrophila* (**a**, **b**) *Bacillus cereus* (**c**, **d**), *Staphylococcus aureus* (**e**, **f**). The left and right sides of plate represented control (without 5-ALA) and experiment group (with 1% or 0.25% 5-ALA)

As shown in Fig. 5a–f, the 100% killing rates were achieved with 1% and 0.25% of 5-ALA for *A. hydrophila*, *B. cereus* or *S. aureus*, though the optimal 5-ALA concentration was 3% for *P. hauseri* (Table 3). The four harmful pathogens were eliminated by 5-ALA-aPDT, verifying the feasibility of purified 5-ALA, which showed impressive antibacterial activity against pathogens as pure 5-ALA from commercial.

### Growth improvement and *A. hydrophila* elimination in algae culture

Microalgae is an innovative biofuel producer in the next-generation bioenergy. However, scaling-up of microalgae in the open-pond is still challenge due to bacterial contamination (Wang et al. 2013). Currently, chemical pesticides are extensively used to protect the microalgae system, such as trichlorfon, methyl parathion, and diazinon (Zhu et al. 2020). Although the chemical control showed effective prevention of contamination in microalgae culture, the reagents would inhibit the growth of microalgae (Chen and Jiang 2011). 5-ALA has been applied to kill bacteria and improve the plant growth (Wang et al. 2021), as well as trigger tolerance to cold, high salt conditions (Zhang et al. 2006), and enhance pigment production (Lyu et al. 2022). Therefore, 5-ALA is a promising natural compound to resolve the biological contamination of microalgae.

**Fig. 6:**
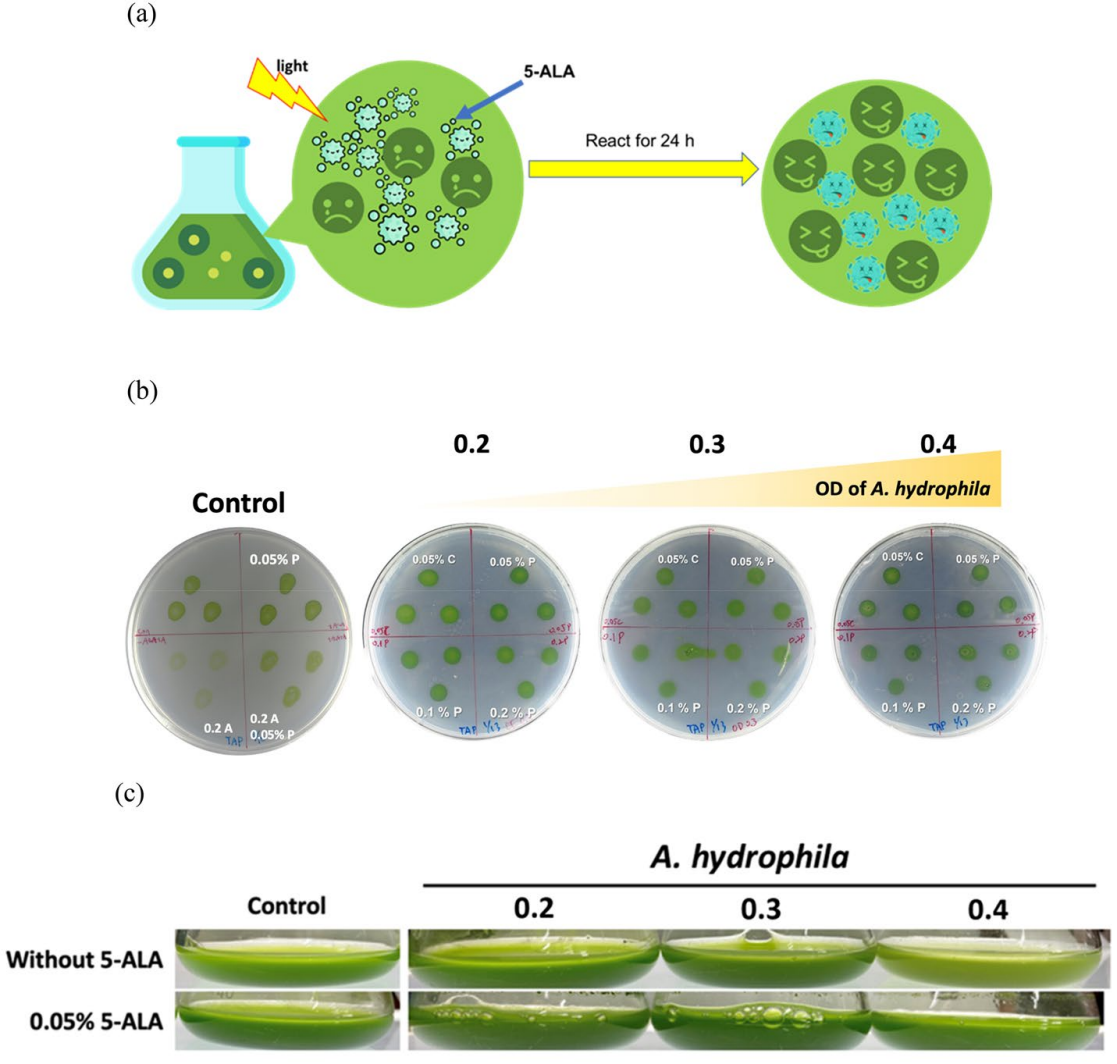
**(a)** Schematic of experiment design for 5-ALA against A. hydrophila, a major fish and algae pathogen. (**b)** The antibacterial activity on TAP plate assay with different concentrations of 5-ALA against different OD_600nm_ of *A. hydrophila* in microalgae *C. sorokiniana* culture at 30 °C for 3 days under 100 µmol/m^2^/s light intensity. C indicates the commercial 5-ALA from Sigma (Sigma A3785) and P is the purified 5-ALA in this study. (**c)** The cultivation of *C. sorokiniana* with 0.05% or without 5-ALA and different biomass of *A. hydrophila* in terms of OD_600_ at 0.2, 0.3 and 0.4 after 3-day culture

The schematic diagram of 5-ALA-aPDT in algae–bacteria co-culture (AB culture) is shown as Fig. 6a. To confirm the effectiveness of 5-ALA-aPDT against *A. hydrophila* which is a famous pathogen in aquatic condition for Cs culture, 0.05%, 0.1% or 0.2% 5-ALA were applied to the AB culture. The growth of Cs was inhibited with *A. hydrophila* without 5-ALA (Fig. 6b, 0.2A) compared to the control strain, while the growth was improved with 0.05% 5-ALA in the absence of *A. hydrophila* (Fig. 6b, 0.05% P). Moreover, the cultivation of Cs showed better growth with 5-ALA and lower concentration of *A. hydrophila* (Fig. 6c), ascertaining the elevation of cell growth with 5-ALA (Jiao et al. 2017). Notably, the 5-ALA concentration should be increased when more *A. hydrophila* cell was added (Fig. 6c). The results showed the outstanding performance of purified 5-ALA to eliminate pathogen in AB culture.

## Conclusion

Due to the imperative demand of 5-ALA, the production and purification is exigent in recent years. Herein, a 92% recovery of 5-ALA from ion-exchange chromatography was achieved by 1 M ammonia at pH 9.5. The activated carbon was applied to remove the pigments in the culture broth. After being concentrated 5-ALA to 500 g/L, the poor solvent method was executed for precipitation, obtaining 50% purity from acetone. The purified 5-ALA was implemented to cancer cells and pathogens elimination, achieving 83% and 100% efficiency, respectively. Moreover, the cell growth of algae *Chlorella sorokiniana* was enhanced while the pathogen, *A. hydrophila*, was killed in the co-culture system using aPDT. A practical and feasible purification and the advantages of 5-ALA in various applications were demonstrated in this study.

## Data Availability

The authors approved the availability of data and materials for publishing the manuscript.

## References

[CR1] Armbruster CE, Mobley HL (2012). Merging mythology and morphology: the multifaceted lifestyle of *Proteus mirabilis*. Nat Rev Microbiol.

[CR2] Bunke A, Zerbe O, Schmid H, Burmeister G, Merkle HP, Gander B (2000). Degradation mechanism and stability of 5-aminolevulinic acid. J Pharm Sci.

[CR3] Cai J, Zheng Q, Huang H, Li B (2018). 5-aminolevulinic acid mediated photodynamic therapy inhibits survival activity and promotes apoptosis of A375 and A431 cells. Photodiagnosis Photodyn Ther.

[CR4] Chen H, Jiang JG (2011). Toxic effects of chemical pesticides (trichlorfon and dimehypo) on *Dunaliella salina*. Chemosphere.

[CR5] Di Venosa G, Fukuda H, Perotti C, Batlle A, Casas A (2004). A method for separating ALA from ALA derivatives using ionic exchange extraction. J Photochem Photobiol B.

[CR6] Din NAS, Lim SJ, Maskat MY, Abd Mutalib S, Zaini NAM (2021). Lactic acid separation and recovery from fermentation broth by ion-exchange resin: a review. Bioresour Bioprocess.

[CR7] Hotta Y, Tanaka T, Takaoka H, Takeuchi Y, Konnai M (1997). Promotive effects of 5-aminolevulinic acid on the yield of several crops. Plant Growth Regul.

[CR8] Inoue K (2017). 5-Aminolevulinic acid-mediated photodynamic therapy for bladder cancer. Int J Urol.

[CR9] Jiao K, Chang J, Zeng X, Ng IS, Xiao Z, Sun Y, Lin L (2017). 5-Aminolevulinic acid promotes arachidonic acid biosynthesis in the red microalga *Porphyridium purpureum*. Biotechnol Biofuels.

[CR10] Juzeniene A, Juzenas P, Iani V, Moan J (2002). Topical application of 5-aminolevulinic acid and its methylester, hexylester and octylester derivatives: considerations for dosimetry in mouse skin model¶. Photochem Photobiol.

[CR11] Kennedy J, Pottier RH, Pross DC (1990). Photodynamic therapy with endogenous protoporphyrin: IX: basic principles and present clinical experience. J Photochem Photobiol B.

[CR12] Klausen M, Ucuncu M, Bradley M (2020). Design of photosensitizing agents for targeted antimicrobial photodynamic therapy. Molecules.

[CR13] Kussovski V, Mantareva V, Angelov I, Orozova P, Wöhrle D, Schnurpfeil G, Avramov L (2009). Photodynamic inactivation of *Aeromonas hydrophila* by cationic phthalocyanines with different hydrophobicity. FEMS Microbiol Lett.

[CR14] Lin JY, Xue C, Tan SI, Ng IS (2021). Pyridoxal kinase PdxY mediated carbon dioxide assimilation to enhance the biomass in *Chlamydomonas reinhardtii* CC-400. Bioresour Technol.

[CR15] Lyu X, Lyu Y, Yu H, Chen W, Ye L, Yang R (2022). Biotechnological advances for improving natural pigment production: a state-of-the-art review. Bioresour Bioprocess.

[CR16] Matsumura Y, Takeshima YI, Okita H (1994). A convenient method for introducing oxo group into the β-position of cyclic amines and its application to synthesis of δ-aminolevulinic acid. Bull Chem Soc Jpn.

[CR17] Miyachi N, Tanaka T, Nishikawa S, Takeya H, Hotta Y (1998). Preparation and chemical properties of 5-aminolevulinic acid and its derivatives. Porphyrins.

[CR18] Moreira MJA, Gando-Ferreira LM (2012). Separation of phenylalanine and tyrosine by ion-exchange using a strong-base anionic resin. II Cyclic Adsorption/desorption Studies. Biochem Eng J.

[CR19] Okada H, Tanaka T, Nomura T (2016) Method for producing 5-aminolevulinic acid hydrochloride. EP Patent 1,927,586, 14 Apr 2016.

[CR20] Patrickios CS, Yamasaki EN (1995). Polypeptide amino acid composition and isoelectric point ii. comparison between experiment and theory. Anal Biochem.

[CR21] Pérez-Laguna V, García-Luque I, Ballesta S, Pérez-Artiaga L, Lampaya-Pérez V, Samper S, Gilaberte Y (2018). Antimicrobial photodynamic activity of Rose Bengal, alone or in combination with Gentamicin, against planktonic and biofilm *Staphylococcus aureus*. Photodiagnosis Photodyn Ther.

[CR22] do Prado-Silva L, Alvarenga VO, Braga GÚ, Sant’Ana AS (2021). Inactivation kinetics of *Bacillus cereus* vegetative cells and spores from different sources by antimicrobial photodynamic treatment (aPDT). LWT-Food Sci Technol.

[CR23] Sarmah P, Dan MM, Adapa D, Sarangi TK (2018). A review on common pathogenic microorganisms and their impact on human health. Electron J Biol.

[CR24] Sasaki K, Watanabe M, Tanaka T (2002). Biosynthesis, biotechnological production and applications of 5-aminolevulinic acid. Appl Microbiol Biotechnol.

[CR25] Shukla SK, Sharma AK, Gupta V, Kalonia A, Shaw P (2020). Challenges with wound infection models in drug development. Curr Drug Targets.

[CR26] Simões D, Miguel SP, Ribeiro MP, Coutinho P, Mendonça AG, Correia IJ (2018). Recent advances on antimicrobial wound dressing: A review. Eur J Pharm Biopharm.

[CR27] Tachiya N (2016) Novel crystal of 5-aminolevulinic acid phosphate and process for production thereof. EP Patent 2,053,039, 16 Mar 2016.

[CR28] Teijo MJ, Diez BA, Battle A, Fukuda H (2016). Modulation of 5-Aminolevulinic acid mediated photodynamic therapy induced cell death in a human lung adenocarcinoma cell line. Integr Cancer Sci Ther.

[CR29] Tripetch P, Srzednicki G, Borompichaichartkul C (2013). Separation process of 5-aminolevulinic acid from *Rhodobacter spaeroides* for increasing value of agricultural product by ion exchange chromatography. Acta Hort.

[CR30] Wachowska M, Muchowicz A, Firczuk M, Gabrysiak M, Winiarska M, Wańczyk M, Golab J (2011). Aminolevulinic acid (ALA) as a prodrug in photodynamic therapy of cancer. Molecules.

[CR31] Wang H, Zhang W, Chen L, Wang J, Liu T (2013). The contamination and control of biological pollutants in mass cultivation of microalgae. Bioresour Technol.

[CR32] Wang J, Zhang J, Li J, Dawuda MM, Ali B, Wu Y, Xie J (2021). Exogenous application of 5-aminolevulinic acid promotes coloration and improves the quality of tomato fruit by regulating carotenoid metabolism. Front Plant Sci.

[CR33] Xue C, Hsu KM, Ting WW, Huang SF, Lin HY, Li SF, Ng IS (2020). Efficient biotransformation of L-lysine into cadaverine by strengthening pyridoxal 5’-phosphate-dependent proteins in *Escherichia coli* with cold shock treatment. Biochem Eng J.

[CR34] Yi YC, Shih IT, Yu TH, Lee YJ, Ng IS (2021). Challenges and opportunities of bioprocessing 5-aminolevulinic acid using genetic and metabolic engineering: a critical review. Bioresour Bioprocess.

[CR35] Yi YC, Xue C, Ng IS (2021). Low-carbon-footprint production of high-end 5-aminolevulinic acid via integrative strain engineering and rubisco-equipped *Escherichia coli*. ACS Sustain Chem Eng.

[CR36] Yu TH, Tan SI, Yi YC, Xue C, Ting WW, Chang JJ, Ng IS (2022). New insight into the codon usage and medium optimization toward stable and high-level 5-aminolevulinic acid production in *Escherichia coli*. Biochem Eng J.

[CR37] Zhang ZJ, Li HZ, Zhou WJ, Takeuchi Y, Yoneyama K (2006). Effect of 5-aminolevulinic acid on development and salt tolerance of potato (*Solanum tuberosum L.*) microtubers in vitro. Plant Growth Regul.

[CR38] Zhu Z, Jiang J, Fa Y (2020). Overcoming the biological contamination in microalgae and cyanobacteria mass cultivations for photosynthetic biofuel production. Molecules.

